# Generative artificial intelligence in neurology: Opportunities and risks

**DOI:** 10.1111/ene.16232

**Published:** 2024-02-08

**Authors:** Antonio Cerasa, Byron Crowe

**Affiliations:** ^1^ Institute for Biomedical Research and Innovation National Research Council of Italy Messina Italy; ^2^ S'Anna Institute Crotone Italy; ^3^ Pharmacotechnology Documentation and Transfer Unit, Preclinical and Translational Pharmacology, Department of Pharmacy, Health Science, and Nutrition University of Calabria Rende Italy; ^4^ Department of Medicine Beth Israel Deaconess Medical Center Boston Massachusetts USA; ^5^ Harvard Medical School Boston Massachusetts USA

Dear Editor,

In this issue of the journal, Fonseca et al. [1] present results of a creative analysis comparing performance between human neurologists and a generative artificial intelligence (AI) on neurology boards‐style questions. The authors utilized OpenAI's AI model Generative Pre‐Trained Transformer 3.5 (GPT 3.5) and challenged it with a set of 188 questions pooled from the American Academy of Neurology's “Question of the Day” application. About 1500 neurologists worldwide respond to these daily questions and the percentage of correct responses are reported, allowing the authors to compare GPT 3.5 performance to that of a global community of neurologists. As anticipated, the AI achieved impressive scores, answering 71.3% of questions correctly compared to 69.2% for neurologists. Moreover, the authors reviewed the AI justification for each response and found it was appropriate 96.1% of the time. Importantly, this study has a key nuance in that it utilized a slightly older AI model, GPT 3.5, rather than current state‐of‐the art model, GPT‐4. Performance difference with GPT‐4 is notably higher than GPT 3.5 on medical questions, with GPT‐4 achieving greater than 90% accuracy on the medical knowledge benchmarking test MedQA compared to only 60% for GPT 3.5 [2]. Point being, it is most likely that GPT‐4 can achieve even better results than those observed in the current study.

The staggering growth and pace of development of generative AI tools like ChatGPT since late 2022 is expected to impact the health care sector. The exceptional natural language processing skills of these models allow them to not only correctly answer multiple‐choice medical questions but perform extremely well on free‐text clinical reasoning [3] and analysis of complex cases [4]. Strong et al. [3] found that GPT‐4 performed better than medical students and achieved an overall score of 93% on free‐text clinical reasoning responses to standardized cases, and Kanjee et al. [5] found that GPT‐4 was able to surface the correct diagnosis in 64% of challenging diagnostic cases sourced from *New England Journal of Medicine* clinicopathological case conferences. Although real‐world diagnostic performance of the newest generative AI models like GPT‐4 remains forthcoming, AI chatbots powered by machine learning algorithms have previously shown the ability to achieve high concordance with physician diagnosis in real‐world settings, with an AI achieving greater than 95% agreement with the physician diagnoses in nearly half of cases presenting to a virtual primary care clinic [6]. These early results point to a new era in which generative AI will provide substantial support to physicians throughout the clinical decision‐making process.

However, before generative AI can enter meaningfully into neurological practice, more work must be done to understand where it can augment human intelligence, and where it falls short. Generative AI models encode large amounts of clinical knowledge and can additionally reference external sources, raising the possibility of a generalized diagnostic and treatment selection tool. The adoption of health care AI to augment human intelligence has precedent; for example, AI‐based algorithms joined clinical neurology around 2005–2010, first being applied to omics data for accurate automatic classifications of neurological patients. However, adoption of generative AI tools faces a different barrier, as these tools replicate clinical reasoning, previously the exclusive domain of the expert physician. Unlike multiple‐choice questions, real‐world clinical “reasoning” involves the collection of relevant data, interpreting key details and nuance, and making diagnostic and treatment decisions that integrate the totality of evidence and unique patient circumstances. Generative AI models like GPT‐4 reach an answer by leveraging an incredibly large neural network architecture with word associations. Although evidence provided by Fonseca et al. [1], Kung et al. [4], and Kanjee et al. [5] confirms that AI uses accurate information to generate its answers, it cannot practice the "art of medicine," that unique synthesis of technical medical knowledge with the myriad human factors that influence judgment and decision‐making.

Obviously, generative AI has limitations. First, it is important to remember that generative AI requires domain‐specific knowledge to be most effective; therefore, targeted analyses of areas within the field of neurology are preferable. Finally, real‐world studies are needed to understand how clinicians interact with generative AI. These tools are not panaceas, but have strengths and weaknesses that must be better understood in the context of actual implementations and human–computer interaction, not just knowledge benchmarking.

Generative AI is a clearly powerful technology, and early evidence continues to demonstrate its potential to completely transform major domains of clinical practice. Through better automation and personalization of the care process, generative AI may help patients receive more tailored guidance on their condition while offloading many of these tasks from the physician to the AI, preserving expert time for the most challenging and nuanced cases. Finally, we agree with Clusmann et al. [7] that AI‐related algorithms will give us the opportunity to democratize access to scientific evidence and to excellent health care services.

## AUTHOR CONTRIBUTIONS

**Antonio Cerasa:** Conceptualization; writing – original draft; supervision. **Byron Crowe:** Conceptualization; supervision; methodology; writing – review and editing.

## CONFLICT OF INTEREST STATEMENT

A.C. and B.C. declare no conflicts of interest.

## Data Availability

The data that support the findings of this study are available on request from the corresponding author. The data are not publicly available due to privacy or ethical restrictions.
